# The efficacy of varenicline in achieving abstinence among waterpipe tobacco smokers – study protocol for a randomized controlled trial

**DOI:** 10.1186/s13063-016-1761-y

**Published:** 2017-01-11

**Authors:** Raana Zahid, Omara Dogar, Sonia Mansoor, Amina Khan, Mona Kanaan, Mohammed Jawad, Jasjit S. Ahluwalia, Kamran Siddiqi

**Affiliations:** 1 The Initiative, Islamabad, Pakistan; 2 Orange Grove Farm, Korung Road, Banigala, Islamabad, Pakistan; 3 Department of Health Sciences, University of York, Seebohm Rowntree Building, Heslington, York, YO10 5DD UK; 4 Department of Health Sciences, University of York, ARRC Building, Heslington, York, YO10 5DD UK; 5 Department of Primary Care and Public Health, Imperial College London, Hammersmith, W6 8RP UK; 6 Milltown, NJ USA

**Keywords:** Waterpipe, Tobacco, Smoking, Hookah, Shisha, Pharmacotherapy, Varenicline, Cessation, Abstinence, Behavioral support

## Abstract

**Background:**

Waterpipe tobacco smoking has increased among youth across the globe including in the US, and it continues as a common and traditional form of smoking tobacco in Pakistan. A range of behavioral and pharmacological therapies are available to support people in quitting cigarette smoking; however, little evidence exists for the efficacy of these therapies in achieving abstinence among waterpipe tobacco smokers. The objective of this study is to assess the efficacy of varenicline when added to behavioral support for waterpipe tobacco smoking cessation, by measuring biochemically validated continuous abstinence in waterpipe tobacco smokers.

**Methods/design:**

This is a two-arm, double-blind, placebo-controlled randomized trial conducted in four districts in Punjab, Pakistan. Study participants include adults using a waterpipe (with or without concomitant cigarette, bidi or other forms of tobacco smoking) on a daily basis for at least 6 months and who are willing to quit. We will individually randomize 510 participants to one of the two arms of the trial. Participants in the intervention arm will receive varenicline and behavioral support and those in the control arm will receive placebo and behavioral support. The primary outcome will be continuous abstinence for at least 6 months (week 25) which is biochemically verified by a carbon monoxide level of <10 ppm. Secondary outcomes will include biochemically verified 7-day point abstinence at 5, 12 and 25 weeks and any lapses and relapses between the different assessment points. Tertiary outcomes will include assessment of withdrawal symptoms using the Mood and Physical Symptoms Scale (MPSS), smoking dependency using the Lebanon Waterpipe Dependency Scale (LWDS-11) and monitoring adverse outcomes.

**Discussion:**

This is an efficacy trial and would require a subsequent effectiveness trial for a definitive evaluation of the intervention.

**Trial registration:**

ISRCTN, ISRCTN94103375. Registered on 1 December 2015.

**Electronic supplementary material:**

The online version of this article (doi:10.1186/s13063-016-1761-y) contains supplementary material, which is available to authorized users.

## Background

Waterpipe tobacco smoking, also known as hookah, Shisha or narghile, is a growing public health concern worldwide. In waterpipe tobacco smoking, tobacco is heated by burning charcoal in a stemmed, water-containing apparatus. Users inhale a mixture of tobacco and charcoal smoke by breathing in on a hose that is attached to the apparatus. One waterpipe session can last between several minutes to several hours [1]. The activity has been practiced for centuries and, while popularity has markedly increased worldwide, it remains embedded within south Asian culture, particularly in Pakistan. According to the latest Global Adult Tobacco Survey (GATS, 2014) conducted in Pakistan, 22.2% of men, 2.1% of women and 12.4% of the overall adult population currently smoke tobacco (15.6 million adults currently smoke tobacco). The same survey states that 4.7% of men, 1.1% of women and 3.0% of the overall adult population currently use a waterpipe (3.7 million adults). Furthermore, the GATS, 2014 states that about one in four smokers made an attempt to quit in the past 12 months. Despite the widely held belief that waterpipe tobacco smoking is safer than cigarette smoking [2], research has shown the contrary. The tobacco mixture contains significant levels of nicotine, known for its addictive properties, as well as tobacco-specific nitrosamines, volatile aldehydes, heavy metals and “tar,” which cause respiratory diseases and cancer [3, 4]. Tobacco-like adverse health effects from waterpipe tobacco smoking are, therefore, expected and well-documented in a recent meta-analysis, which revealed a positive association between waterpipe tobacco smoking and lung (odds ratio (OR) 4.6, 95% CI 2.6–8.0) and esophageal (OR 3.6, 95% CI 1.4–9.4) cancers [5]. As with cigarettes, nicotine dependence is a key feature of regular waterpipe tobacco smokers who exhibit cravings, withdrawal symptoms and other nicotine-modulated behaviors [6, 7].

The effectiveness of smoking-cessation strategies including behavioral support and pharmacotherapies, such as varenicline, has been well-established among cigarette smokers [8, 9]. Varenicline has also shown to be more effective and cost-effective than nicotine replacement therapy [10] and bupropion [11], respectively. In a previous study [12] behavioral support was found to be effective among hookah smokers (relative risk (RR) 2.2; 95% CI 1.3–3.8) but to a lesser extent than among cigarette smokers (RR 5.8; 95% CI 4.0–8.5). This study was a subgroup analysis of a large cluster randomized controlled trial (RCT) (Action to Stop Smoking In Suspected Tuberculosis) in Pakistan, which found 41.0% smoking abstinence in the behavioral support group compared to 8.5% in the control group [13]. Combining varenicline with behavioral support has further potential in increasing cessation success [14]. However, the efficacy of varenicline (either alone or in combination with other therapies) in waterpipe tobacco smoking cessation is currently unknown. The two RCTs to date assessing the effectiveness of smoking cessation strategies in waterpipe tobacco smokers did not evaluate varenicline [12, 15]. Moreover, these trials have either shown little or modest success in getting waterpipe tobacco smokers to quit as compared to cigarette smokers [12, 15]. This calls for further therapies, such as varenicline, to be evaluated in this population. This study will be the first to explore the efficacy of varenicline in waterpipe tobacco smoking cessation. Given the increased use of the waterpipe use across all continents and the paucity of evidence on interventions for its cessation, this study is critically important for public health and could provide a scientific breakthrough in this area. The items in this protocol comply with the recommended SPIRIT checklist (Additional file 1).

## Methods/design

### Aims and objectives

Our key aim is to assess whether varenicline coadministered with behavioral support is more efficacious in achieving 6 months’ continuous abstinence from all forms of tobacco smoking among waterpipe tobacco smokers than a combination of placebo and behavioral support. Therefore, the primary objective of the trial is to assess the efficacy of varenicline when added to behavioral support for smoking cessation, by measuring biochemically validated continuous abstinence at week 25 in waterpipe tobacco smokers. The secondary objectives are to: (1) assess the efficacy of varenicline when given with behavioral support in achieving point abstinence at week 5, week 12 and week 25, (2) compare the efficacy of varenicline when given with behavioral support in achieving point and continuous abstinences between exclusive waterpipe tobacco smokers and those who combine it with other forms of smoking tobacco, (3) assess the proportion of early and late lapses, and their ability to predict abstinent failures and (4) assess the proportion of early and late relapses and determine their predictors. In addition, we will first translate and then assess the psychometric properties of the Mood and Physical Symptoms Scale (MPSS) and the Lebanon Waterpipe Dependency Scale (LWDS-11) in the target population.

### Design

This is a two-arm, double-blind, placebo-controlled, randomized trial. Participants randomized to treatment (arm 1) will receive varenicline while those randomized to the control arm (arm 2) will receive placebo. In addition, behavioral support will be provided to participants in both arms.

### Settings

The study is being conducted in four districts (Chakwal, Khushab, MandiBahauddin, and Rawalpindi) of Punjab, Pakistan. Based in district hospitals in three districts and a teaching hospital in one district (Rawalpindi), the study will recruit participants from a large catchment population in both urban and rural settings.

### Study participants

We will recruit 510 adults who smoke a waterpipe on a daily basis for at least 6 months, with or without concomitant cigarette, bidi or other tobacco smoking and who wish to quit smoking. We define daily waterpipe tobacco smoking if a person smokes on more than 25 days in a month. We will exclude those who (1) have used any pharmacotherapy for tobacco dependence (including nicotine replacement therapy and electronic cigarettes) in the last 30 days, (2) are pregnant, lactating or planning to become pregnant, (3) have a medical condition requiring hospitalization, (4) have previously used varenicline and had an allergic reaction, (5) have a history of heart disease, including unstable angina, untreated cardiac arrhythmia, myocardial infarction, or have undergone a cardiac procedure (in the last 3 months), (6) have uncontrolled hypertension or a history of stroke, (7) have a history of chronic kidney disease, (8) have a history of epilepsy, (9) have suicidal ideation or a history of self-harm, (10) have a history of schizophrenia, psychosis or bipolar disorder, (11) have current moderate or severe depression, (12) currently use smokeless tobacco and (13) actively use substances (including alcohol misuse) other than tobacco.

The following methods will be used to identify and recruit potential participants:

1. Recruiting hospital patients: we will provide half-day training to all health care staff in each hospital to identify potential trial participants among adult hospital attendees and refer them to a research assistant based in a respective hospital for eligibility assessment
2. Recruiting patients’ relatives/attendants: we will put up posters and make leaflets available in the reception hall, outpatient departments, hospital wards and other relevant departments with a brief explanation about the study and inviting participants. These will be targeted at patients as well as their attendants. Interested people will be asked to contact a respective research assistant
3. Recruiting members of public: we will also recruit eligible and consenting individuals among catchment populations of the participating hospitals. We will advertise our trial and invite people to participate through local newspapers, local cable TV and/or using community networks. Interested people will be also identified through local connections and/or previously enrolled participants in the trial and put in contact with the research assistant. Individuals recruited via community networks will be offered trial enrollment in the community in case they are not able to visit the respective hospital site

Once identified and referred to our resident research assistant, all potential participants will be assessed for eligibility. Research assistants will be trained to go through the inclusion and exclusion criteria and make a final eligibility assessment. A screening register will be kept at each site, which will have potential participants’ “screening” number, responses to questions asked to assess eligibility and the outcome of their eligibility assessment. Those found to be eligible will be given verbal and written information about the trial and 24 h to consider participation. Potential participants will be given an opportunity to clarify anything that they do not understand and ask related questions. It will also be explained that they are free to leave the study at any point without any consequences on their routine and entitled medical treatment. Participants will not receive any financial incentives to participate in the trial except to cover their travel expenses. Written consent will be obtained by the research assistant from those interested by going through a checklist on the Consent Form and obtaining an ink signature or a thumb impression (thumb impression is officially acceptable in Pakistan for those who cannot write). The outcome on the Consent Form will also be recorded on the eligibility register. All eligible and consenting participants will be formally enrolled in the trial and a minimum set of information (sex, concomitant smoking other than waterpipe) will be obtained, necessary for randomization. Where applicable, reasons for not meeting the eligibility criteria or declining to participate will be recorded on the screening register.

Treatment can be withdrawn at any time after randomization and allocation if significant intolerance to the study treatment is suspected. Other reasons for withdrawing from study treatment are: (1) the participant makes a voluntary decision to withdraw from the study, (2) the participant has a serious clinical adverse event, develops a new medical condition or suffers from worsening of any existing illness, which indicates that continuing in the study will not be in their best interest. Study treatment will also be withdrawn if the participant develops a life-threatening or severely disabling medical condition, or requires hospitalization and (3) female participants who become pregnant or intend to become pregnant. If a participant is withdrawn from the study due to treatment intolerance or for any of the above reasons, their follow-up assessments and data collection will continue as per protocol. If the treatment is discontinued due to drug intolerance or any serious clinical adverse event, the participant will be followed up until the intolerance/event subsides and there is a return to an acceptable clinical status, ascertained by a physician.

#### Randomization and allocation

Those who consent will be randomly assigned to one of the two treatment conditions by using a computer-generated allocation sequence designed at the University of York. The system, created in software R v3.2.2, will generate a permuted block randomization list for each site with stratification factors including gender and concomitant smoking. Based on this random sequence, the system will allocate each newly recruited participant either to varenicline or to placebo treatment. For treatment allocation, the research assistant will make a phone call to the trial manager based at the central research office in Islamabad. On providing the basic information on recruiting district, gender, and concomitant smoking, the trial manager will generate a trial ID by running a prespecified code (for each random block) in the R file. At this point, both the trial manager and the research assistant will be unaware of the treatment condition associated with each trial ID. The trial ID will correspond to that on the medication packs already made available at each participating hospital. Under no circumstances will an enrolled participant be dispensed a medication pack other than the one assigned through the randomization system. To ensure double-blinding, we will use identical medication packs for both placebo and varenicline, labeled only with a unique trial ID. The investigators, research assistants and participants will be blinded to the allocation until the trial database is locked at the end of the study.

### Interventions

Once enrolled, participants in the trial will be randomized to receive behavioral support either with varenicline or with placebo.

### Behavioral support

Behavioral support will be offered to all waterpipe tobacco smokers as part of routine care. Hence, all trial participants, irrespective of their treatment condition, will receive behavioral support intervention using an educational flipbook. This will consist of two structured sessions. A 30-min session at the first visit will aim to encouraging waterpipe tobacco smokers to see themselves as nonusers and to set a plan for a quit day 1 week later. This is then followed by a 10-min session, coinciding with their quit day, to review progress. Further encouragement and support will be offered at subsequent visits in week 5.

### Varenicline

Participants allocated to the varenicline and behavioral support arm will receive their first week’s supply on the day of trial enrollment. The treatment, in the form of 0.5-mg tablets, will be dosed at 0.5 mg once daily on days 1–3 and 0.5 mg twice daily on days 4–7. Participants will be expected to return at the end of week 1, coinciding with their quit day, at which point they will be dispensed another pack of medication for 11 weeks, with the pill in the form of 1-mg tablets to be taken twice daily for the rest of the treatment duration. Adherence will be monitored at each visit by using a 7-day timeline follow-back and by looking at the tablets left in the bottle. A 7-day timeline follow-back will consist of a set of questions administered at weeks 1, 5 and 12 asking participants if they have taken the prescribed treatment in the previous week by recalling each day of the week. Adherence will be considered “complete” if the participant adheres throughout 12 weeks; and “partial” if adherence is either irregular or not for the entire period. Participants will be asked to record the nature, timing and duration of any adverse events (AEs) with clear guidance on when to stop the medication and when, and how, to report back to the named clinician in the participating hospital. These clinicians would have received the basic training required in this regard.

### Placebo

By virtue of being a double-blind trial, participants allocated to the placebo and behavioral support arm will be dispensed placebo in exactly the same manner as described above for varenicline, i.e., a pack of 0.5-mg tablets in the first week and a 1-mg pack for the following 11 weeks.

Pfizer is responsible for ensuring that the quality and quantity of the study treatment is adequate for the trial. All treatment packs were shipped to the central research office in Islamabad and the trial manager is responsible for storing these at room temperature. An independent researcher was employed to give the treatment packs their allocation IDs. A list of these trial IDs will be forwarded to the trial statistician who will feed this into the computer program that will generate a random allocation sequence. The treatment packs will then be given out to each hospital where these will be stored in a locked cupboard at room temperature within the hospital pharmacy. Although medication packs will not disclose the treatment condition, their labels will contain the following information: (1) LOT number given by Pfizer, (2) unique trial ID given by an independent researcher, (3) principle investigator’s details, (4) sponsor’s details, (5) hospital address, (6) expiry date, (7) patient’s name and (8) directions for use. Each pack will also clearly say “For use in Hookah Trial only.” A treatment supply register will be kept both at the hospital as well as at the central research office. All left-over treatment packs will be returned from the participating hospital sites to the central research office and will be disposed off after completion of the trial follow-ups.

### Primary outcome

As per Russell’s Standard [16], the primary outcome will be self-reported continuous abstinence for at least 6 months (no smoking allowed in the 7 days prior to each of the three assessments) which is biochemically verified by a carbon monoxide (CO) level of <10 ppm measured by Micro CO (Micro Medical Ltd., Rochester, United Kingdom) at week 5, week 12 and week 25. When a participant self-reports abstinence with an elevated CO level of >10 ppm on any of the three assessments, we will use urinary kits for checking cotinine levels in such cases. Depending on the cotinine level findings the participant will be categorized as a smoker or not.

### Secondary outcomes

Based on the combination of self-report and CO levels, these will include:

1. Point abstinence, defined as a self-report of not smoking in the previous 7 days and verified by a CO level of <10 ppm, at week 5, week 12 and week 25
2. Early lapse, defined by a self-report of smoking (even once) after the quit date but having point abstinence at week 5
3. Late lapse, defined by a self-report of smoking (even once) between week 5 and week 12 but showing point abstinence at week 5 and week 12
4. Early relapse, defined by point abstinence at week 5 but a smoking status in later assessments
5. Late relapse, defined by point abstinence at week 5 and week 12 but a smoking status at week 25
6. Differences in the point and continuous abstinences, lapses and relapses between exclusive waterpipe smokers and those who combine it with other forms of smoking tobacco

We will also translate the MPSS [17] for use in a Pakistani population. Once translated, the scale will be administered at baseline and week 25. The scale assesses withdrawal symptoms including anxiety, depression, irritability, restlessness, hunger, concentration and sleep. It also assesses the frequency and strength of urges to smoke. As above, we will also translate the LWDS-11, a tobacco-dependence measure [7], to be administered at baseline and week 25. The scale consists of 11 items and four subscales, the first representing nicotine dependence, the second negative reinforcement, the third psychological craving and the fourth positive reinforcement.

### Data collection and management (Fig. 1)

**Fig. 1:**
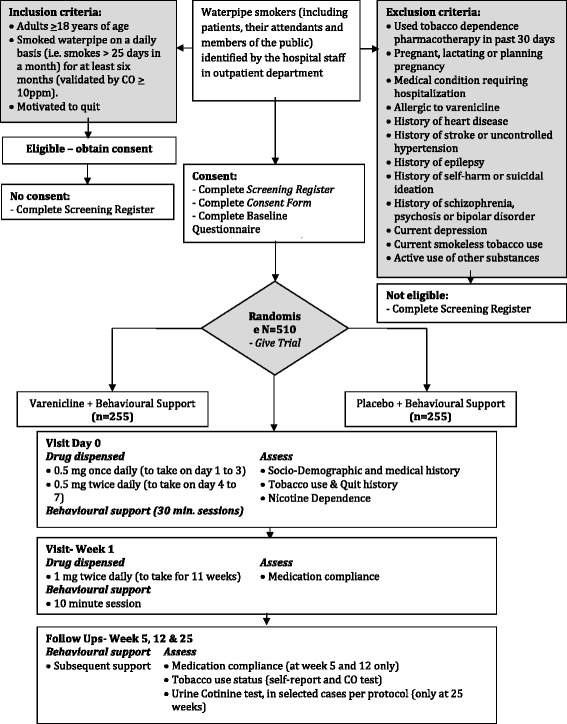
CONSORT Flow Diagram

**Fig. 2:**
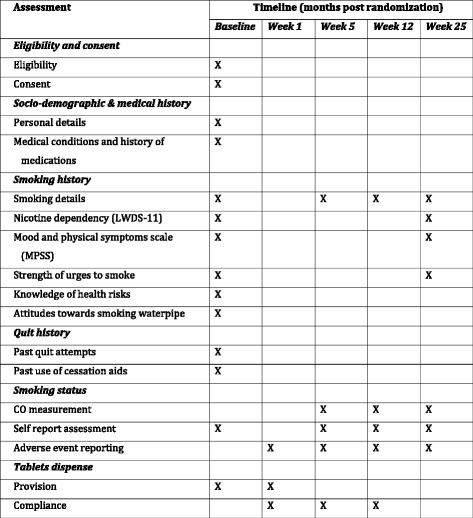
Data collection schedule

All participants will undergo a baseline assessment including demographic variables (e.g., age, sex, ethnicity, socioeconomic status), past and present smoking (all forms), motivation to quit, withdrawal symptoms (MPSS) and dependency assessments (LWDS-11). Information on waterpipe use will also be collected which will include the quantity, duration and frequency of waterpipe tobacco smoked at the baseline. We will also enquire about their knowledge of health risks associated with waterpipe tobacco smoking, attitudes towards waterpipe tobacco smoking, and intention to quit. The use of concomitant medications will also be collected at baseline. Although social support is not directly assessed, whether smoking is permitted inside the home is assessed. Further assessments at weeks 5, 12 and 25 will include current smoking, any changes in smoking patterns, medication-related AEs and CO breath tests. MPSS, LWDS-11, Strength of Urges to Smoke (SUTS) and cotinine urine tests will be carried out at week 25 (Fig. 2). To pay for their travel and subsistence expenses, participants will receive 200 Pakistani Rupees (approximately US$2) at the last follow-up. Those failing to attend will be reminded and asked to at least send their smoking status via text (mobile phone coverage is high in Pakistan). Where possible, a home visit will be arranged to perform CO measurements. In our previous experience of conducting smoking-cessation trials in Pakistan, such strategies have been successful in keeping attrition rates below 10% [13]. Data will still be collected and maintained on those who either discontinue or deviate from treatment protocols to assess fidelity to the treatment. Data will be initially collected in the form of paper-based questionnaires. Containing no participant-identifiable information, these will be kept in a locked cupboard separate from the Consent Forms at the participating hospital. Every week, these will be photocopied and sent over to the central research office through a secure courier service. Once received, data will be entered in a secure trial database managed by the University of York, created using Qualtrics software (Qualtrics, Provo, UT, USA). Paper copies will also be kept secure in a locked cupboard.

### Adverse events

There will be a vigilant surveillance system in place for adverse events (AEs) occurring during the course of the trial with particular emphasis on identifying, recording, reporting and managing serious suspected drug reactions. We will use standard definitions to distinguish between an adverse event (AE), a serious adverse event (SAE) and a serious suspected adverse drug reaction (SSARD).

In the event of any AE reported by the participant, their attendant or health care staff, the research assistant will complete an AE Form, which will include any available medical diagnosis. For reporting SAEs, a form will be provided by Pfizer known as a Pfizer-provided Investigator-initiated Research Serious Adverse Event Form. The Reportable Event Fax Cover Sheet provided by Pfizer will also be included with each SAE submitted. The research assistant will photocopy and complete this form, send it in the post to the central research office and call the trial manager on the same day providing a verbal report of the event. The research assistant will be trained to differentiate between AEs and SAEs. However, the trial manager, who is medically qualified, will ensure that the event is classified appropriately after receiving the verbal report. The trial manager will also code the event using the Medical Dictionary for Regulatory Activities (MedDRA) [18] and cascade the information as follows.

All AEs must be reported to the principal investigator (Pakistan) within 3 days of detection. AE data will be collated and reported to the trial sponsors and National Bioethics Committee at 6-monthly intervals. These must also be reported to the Study Operational Committee and the Independent Trial Steering Committee at their regular meeting. All AEs that have the potential to develop into SAEs will be followed to resolution or stabilization and reported as SAEs if they become serious. All SAEs must be reported to the principal investigator and Pfizer within 24 h of detection and should also be reported to the trial sponsors and the National Bioethics Committee within three working days. All SSARDs should be reported to the sponsors and the committee within 24 h of the event. All serious events must also be reported to all study investigators and the chair of the Independent Trial Steering Committee (within 3 days for SAEs and within 24 h for SSARDs). If the trial manager requires more detail in relation to any SAEs or SSARDs then they may request the trial statistician to unblind the treatment condition. The chief investigator will have the overall responsibility to ensure that all AEs are reported according to the above protocol.

In addition to assessing seriousness, the trial manager, who is medically qualified, will assess all AE for causality, severity and expectedness. This will be done in consultation with the principal investigator and the event will be classified as follows:

- Unrelated: when the event is considered not related to the study treatment
- Possibly: when an association of the event with the study treatment cannot be ruled out
- Probably: when temporal association and an absence of any other explanation suggest that the event could be related to the study treatment
- Definitely: based either on known side effects of the study treatment or on challenge testing, a suggestion that the study treatment is the most likely cause of the event

All AEs/SAEs that fall under the possible, probable or definitive category will be classified as adverse reactions or SSADRs.

The trial manager can make the following assessment based on severity, which should not be confused with seriousness (a statutory definition) differentiating between AEs and SAEs:

- Mild: these events cause minimal discomfort, are easily tolerated and do not interfere with routine life activities
- Moderate: these events cause moderate discomfort and do interfere with routine life activities
- Severe: these events cause much discomfort and lead participants to stop their routine life activities

If the event is judged to be an adverse reaction, serious or otherwise, this must be judged on expectedness based on what is already known on the safety profile of the drug under study. Each participating hospital site will have a named medical practitioner who will be responsible for dealing with any AE that requires medical attention. All medical expenses resulting from such events will be covered by the trial budget. This will not include any elective procedures, operations or admissions planned prior to participating in the trial. In addition to collecting detailed clinical information on the AE Form, other relevant medical information will be collected from hospital. These events will be followed up till resolution or returning to a stable medical state. We will not expect any events that occur after the completion of follow-up to be relevant to the trial and, therefore, no active surveillance will continue beyond trial completion. Nevertheless, any event reported to the trial manger will be recorded and kept in the records along with other trial data.

### Sample size

The primary outcome of the study is continuous abstinence for at least 6 months between week 5 and week 25. Our previous data from a clustered randomized trial in 33 centers in Pakistan [13] with 1955 participants gave an estimate of 37% continuous abstinence at 25 weeks for the behavioral support arm of the trial. A difference of an additional 13 percentage points in the varenicline group is the minimum clinically important difference that we are interested in detecting; this is the median difference reported by the trials which looked at the effectiveness of varenicline and were included in the systematic review by Cahill et al. [8]. In order to detect an absolute difference of 13% in the varenicline plus behavioral support versus the placebo plus behavioral support group, with 80% power and 5% significance, 228 patients would be required per arm. Allowing for a 10% dropout, a total of 508 patients will, therefore, need to be recruited into the study.

### Statistical analysis

A Consolidated Standards of Reporting Trials (CONSORT) diagram (Fig. 1) shows the flow of participants through the trial. Baseline data including demographic variables will be summarized descriptively by trial arm but no formal statistical comparisons will be undertaken. Continuous measures will be reported as means and standard deviations while categorical data will be reported as counts and percentages.

The main analysis will use log-binomial regression for the primary outcome to estimate any difference in risks between the two arms of the study adjusting for baseline data. We will also investigate any potential clustering at the centre level and family/friends’ level and adjust for it in the regression model. A similar approach will be used for the binary secondary outcomes, namely point abstinence at weeks 5, 12 and 25, lapse between weeks 5 and 12, weeks 12 and 25 and weeks 5 and 25. A subgroup analysis of point and continuous abstinence will compare participants with or without concomitant smoking; this analysis will be conducted by exploring whether there is an interaction between the treatment arm and whether someone exclusively smokes a waterpipe or combines it with other forms of tobacco consumption.

The MPSS items will be analyzed individually, summated to give an overall score and scored in three blocks as suggested by West et al. [17]. Similar approaches will be used for the LWDS-11 [7] and SUTS [19] scales. Appropriate regression analysis will be used for each outcome, linear for the summated scores if the assumptions are met, otherwise appropriate measures will be taken, and ordered logistic regression used for the Likert-scale individual items. Analysis of AEs and SAEs will explore whether these differ by treatment arm using chi-square tests. In case of missing data, multiple imputations and appropriate sensitivity analyses will be conducted. As it is likely that more than one variable will have missing data we will use multiple imputations using chained equations (MICE). A minimum of 10 imputations will be performed; however, the final number of imputations will depend on the missing data. We will report the decisions that we make with regard to the number of imputations and the variables we use in the imputations. We will also conduct a sensitivity analysis to explore the implications of the missing-at-random assumption [20, 21]. A significance level of 0.05 will be used for the primary analysis whereas this will be 0.1 for the secondary analyses.

## Discussion

The trial will be conducted to protect the human rights and dignity of the participant as reflected in the 1996 version of the Helsinki Declaration. Participants will not receive any financial inducement to participate in the trial. In order to protect the trial participants, the following provisions will be made/upheld: the trial has been designed to minimize the burden on participants and any foreseeable risk in relation to the intervention involved; the explicit wishes of the participant will be respected including the right to withdraw from the trial at any time; the interest of the participant will prevail over those of science and society; provision will be made for indemnity by the investigator and sponsor. We will deal with key ethical issues in our research as follows:

- We will obtain written consent from all those who are eligible and, after having received trial information (in the local language) and sufficient time (24 h) to consider, are willing to participate. Those unable to sign will be requested to provide a thumb impression on the consent from, a common and acceptable alternative to a signature in Pakistan
- In line with the Data Protection Act and the Research Governance Framework, all data collected will be confidential, being identified with the unique enrollment number only assigned at the beginning of the trial. The research team will maintain the key linking these numbers with the participants’ contact details. Access to the master key register will be limited to researchers via a password-protected database
- All investigators and collaborators at each research site will be required to submit an individual statement declaring any conflicts of interest on a yearly basis reviewed by the Independent Trial Steering Committee
- Access to the participants’ personal details will be restricted to the necessary members of the research team only. Monitors and auditors may also need to access the data. At the end of the trial, data will be securely archived by the University of York for a minimum of 5 years

We have identified following risks associated with this application and strategies to mitigate these:

- Barriers to obtaining favorable opinion from the Ethics Committee: this is unlikely as this study does not pose major risks nor put undue burden on participants
- Delays in obtaining approval from the Ministry of Health in Punjab, Pakistan: we have the support of tobacco leadership in Punjab for this project
- Lack of collaboration between investigators and hospitals: we have secured expression of interest from all four hospitals to minimize this risk
- Staffing recruitment and retention issues: we will ensure that all recruitment paperwork is prepared and posts are advertised in advance
- Risks associated (accidents and violence) with traveling in Pakistan including: Foreign and Commonwealth Office’s (FCO) advice will be followed while making travel arrangements; we will carry out a full risk assessment according to the institutional policy before any travel
- Slow recruitment: our eligibility criteria are fairly broad and recruitment targets realistic based on the existing service workload and our experience in recruiting trial participants; we recruited approximately 2000 participants in a previous smoking-cessation trial in Pakistan
- Loss to follow-up higher than expected: in the previous trial in Pakistan we found less than 10% attrition rate
- Protocol violations: this will be minimized through pilot work, training, supervision, monitoring and quality control
- Serious adverse events (SAEs): a policy on managing SAEs will be developed as part of the protocol
- Errors or inconsistencies in data entry and collation: data management systems and procedures will be standardized and relevant training will be provided; quality controls will be put in place to minimize errors

Being the first RCT of a pharmacological agent for cessation of waterpipe tobacco smoking, the potential impact of this trial is likely to be high as it addresses a key public health concern. To disseminate study findings to the relevant audience, the study results will be published in leading peer-reviewed journals and presented in international public health/tobacco control conferences. The abstract will be published on the University of York official website and learning from the project will be incorporated into health promotion lectures for Master’s students of public health. The National Tobacco Control Cell will be involved at all stages of the project. A project report and a policy brief of the research process and results will be compiled. Events like No Tobacco Day will be utilized to publicize our work through leaflets, policy briefs and community talks.

## Additional file

Additional file 1: SPIRIT Checklist. (PDF 79 kb)

## Abbreviations

AE: Adverse event
CO: Carbon monoxide
CONSORT: Consolidated Standards of Reporting Trials
FCO: Foreign and Commonwealth Office
LWDS: Lebanon Waterpipe Dependency Scale
MedDRA: Medical Dictionary for Regulatory Activities
MPSS: Mood and Physical Symptoms Scale
SAE: Serious adverse event
SSARD: Serious suspected adverse drug reaction
SUTS: Strength of Urges to Smoke
